# External validation of Standardized KELIM and platinum-resistant recurrence scores in patients with advanced epithelial ovarian cancer

**DOI:** 10.1186/s13048-024-01476-3

**Published:** 2024-07-22

**Authors:** Nina Oufkir, Roman Rouzier, Xavier Paoletti, Claire Bonneau

**Affiliations:** 1 Institut Curie, Inserm U900 - Bioinformatics, Biostatistics, Epidemiology and Computational Systems. Cancer Biology, 35, Rue Dailly, 92210 Saint-Cloud, France; 2 Department of Surgery, Institut Curie, 92210 St Cloud, France; 3 Department of Surgery, Centre François Baclesse, 3, Av. du Général Harris , 14000 Caen, France

**Keywords:** Antigen, CA-125, Kinetics, Ovarian neoplasms, Models, Theoretical, Prognosis, Real world

## Abstract

**Background:**

Neoadjuvant chemotherapy followed by interval debulking surgery is currently a common treatment option for advanced epithelial ovarian cancer (EOC). The Standardized CA-125 ELIMination rate constant K (Std KELIM) and the Platinum Resistant Recurrence (PtRR) Score have been proposed as markers of tumor chemosensitivity. The aim of our study was to validate these tools for predicting platinum sensitivity in a real-world population of patients with advanced EOC treated with neoadjuvant chemotherapy.

**Experimental design:**

All patients with advanced EOC treated with neoadjuvant chemotherapy at the Institut Curie between 2000 and 2015 were included. The Std KELIM was calculated with the CA-125 concentrations during the first 100 days of chemotherapy. The predictive value of Std KELIM and PtRR scores for the risk of subsequent PtRR was assessed using receiver operating characteristic (ROC) curve analysis, logistic regression and calibration curve. Kaplan–Meier survival analysis was performed for the treatment-free interval from platinum (TFIp) therapy and overall survival (OS).

**Results:**

Std KELIM data were available for 149 patients. The AUC was 0.67 for PtRR. A low Std KELIM was significantly associated with PtRR (OR = 0.19 (95% CI [0.06, 0.53], *p* = 0.002)) according to the univariate analysis. The calibration curve of the PtRR showed a slight but significant underestimation (*p* = 0.02) of the probability of platinum resistance. Favorable Std KELIM (≥ 1) alone and combined with the completeness of surgery were associated with significantly better survival in terms of TFIp and OS.

**Conclusions:**

Std KELIM is an early prognostic marker of chemosensitivity in a real-life setting complementary to surgical status. It could help the clinician in the early management of patients by identifying those with a worse prognosis.

**Supplementary Information:**

The online version contains supplementary material available at 10.1186/s13048-024-01476-3.

## Introduction

Epithelial ovarian cancer (EOC) is the eighth most common cancer in women worldwide and the fourth leading cause of cancer mortality in women [1]. Three-quarters of patients are diagnosed at an advanced stage, i.e., stage IIIC or IV, according to the International Federation of Gynecology and Obstetrics (FIGO). The prognosis for EOC patients remains poor despite recent therapeutic advances for some subpopulations, with an overall 5-year survival rate of 51% [2].

The initial management of EOC combines optimal cytoreductive surgery and platinum-based chemotherapy in a neoadjuvant (NAC) or adjuvant setting. Vergote [3] and Kehoe [4] reported similar overall survival rates with NAC compared to adjuvant therapy. Platinum-based NAC followed by maximal cytoreductive IDS has become a viable option for the initial management of advanced EOC for patients who cannot undergo immediate complete cytoreduction surgery [5–7]. Previous studies, such as the SCORPION study, have not observed statistically significant differences in survival between patients who underwent NAC and those who underwent primary cytoreductive surgery [7]. This trial, along with others (EORTC55971 and CHORUS), has several limitations, and its results were subsequently criticized. The ongoing TRUST trial (ENGOT ov33/AGO-OVAR OP7) aims to elucidate the most effective treatment regimen for advanced ovarian cancers. A notable advantage of the neoadjuvant chemotherapy (NAC) approach lies in its capacity to evaluate in vivo tumor chemosensitivity. Furthermore, NAC has the potential to increase the probability of achieving complete cytoreduction surgery, a crucial prognostic factor, by reducing the tumor burden before surgical intervention.

Treatment of EOC is generally characterized by a significant initial response to platinum salts, followed by a high rate of recurrence within two years (70–80%). In recent years, however, this has been reassessed with the addition of maintenance therapies such as antiangiogenic agents and poly (ADP-ribose) polymerase (PARP) inhibitors, resulting in a 2-year PFS of 46% in the olaparib and bevacizumab group and 28% in the bevacizumab group in the PAOLA trial [8]. The time to relapse after platinum-based chemotherapy determines platinum sensitivity, guiding subsequent management. A relapse time of less than 6 months after platinum-based chemotherapy signifies platinum resistance, and a relapse time greater than 6 months signifies platinum sensitivity. In patients who are platinum refractory (initial progression while on treatment) or platinum resistant, the median overall survival is 12 months, compared to 3 years in platinum-sensitive patients [9].

Since the fifth Gynecologic Cancer Intergroup GCIG consensus meeting in 2015, this definition of chemosensitivity has been replaced by the treatment-free interval (TFI) to consider this platinum sensitivity as a spectrum and not a binary condition. This definition is intended to consider new innovative therapies, in addition to the TFI from the last platinum dose (TFIp), the TFI from the last nonplatinum therapy (TFInp) and the last biological agent (TFIb). Nevertheless, this 6-month threshold is still widely used in practice. This dichotomy is echoed in the National Comprehensive Cancer Network (NCCN) recommendations on ovarian cancer, which maintain a distinction between platinum-sensitive and platinum-resistant ovarian cancers. The guidelines highlight the essential role of clinical judgment and flexibility in effectively navigating nuanced distinctions when selecting treatment options.

Serum cancer antigen 125 (CA125) is the most widely used tumor marker in daily practice; however, its relevance is controversial, as only 85% of advanced EOCs have elevated CA125 levels. Numerous studies have been conducted on CA125 in the diagnosis and monitoring of EOC; however, the literature remains relatively poor and heterogeneous regarding its role during NAC and the prediction or early assessment of chemo-sensitivity [10–19]. Karamousa's recent study of individual patient data from the Gynecologic Cancer Intergroup (GCIG) meta-analysis showed that serum CA-125 levels at 3 months from the end of the first line of treatment had good predictive ability for overall survival at 24, 36 and 48 months [20]. However, this predictive capacity is achieved after first-line treatment. Recent methods using the kinetics of biological markers and manage to overcome high inter- and intraindividual variability have been developed to provide very early information. Standardized CA-125 ELIMination rate constant K (KELIM), initially developed in the adjuvant setting [21], has been evaluated in the neoadjuvant setting in the CHIVA cohort (NCT01583322, carboplatin ± nintedanib and IDS). Std KELIM was found to be a major independent predictor of survival and platinum-resistant recurrence [11].

The aim of our study was to evaluate the predictive value of neoadjuvant Std KELIM for platinum sensitivity after NAC in a real-life population with advanced EOC.

## Materials and methods

### Patient selection

Patients with EOC were recruited at two sites of the Curie Institut (Paris and Saint-Cloud, France) between 2000 and 2015. The inclusion criteria were as follows: women with advanced FIGO stage (stage IIB to IV) epithelial ovarian cancer, confirmed by pathology, who received platinum-based NAC, ≥ 18 years. The use of KELIM in the neoadjuvant setting was developed in particular in the CHIVA study, which included epithelial ovarian cancers without excluding poor-sensitive histology types such as clear cells or mucinous subtypes. Notably, histological subtype demonstrated non-significance in univariate analysis [11]. We therefore applied the Std KELIM conditions of use, i.e. a population with advanced epithelial ovarian cancer. All patients underwent initial laparotomy or laparoscopic exploration, followed by NAC. Patients were excluded if they were < 18 years old or if the Std KELIM was not calculable. The rate of NAC among the whole cohort of ovarian cancer patients managed at the institution oscillated between 19 and 39%, with no significant trend over the years of management, as determined by the non-parametric Mann–Kendall test (tau = 0.14, *p* = 0.49) (Supplementary Material).

Informed consent with agreement for the use of their clinical and biological data was obtained. The protocol was reviewed and approved by our institutional review data and ethics board [DATA200189].

### Data collection

Age, histological type, FIGO stage (according to the 2014 classification) [22], grade, BRCA status, number of cycles of NAC, type of NAC, neoadjuvant and adjuvant bevacizumab, quality of cytoreduction, chemotherapy response score (CRS), time to recurrence and/or death were collected for each patient.

The completeness of the cytoreduction score was used to evaluate the quality of surgery [23]: CC0, in the absence of macroscopic postoperative residual disease, or not CC0, combining the scores CC1 (< 2.5 mm) CC2 (2.5 mm—2.5 cm) and CC3 (> 2.5 cm).

The concept of platinum resistance has been defined by two criteria: the conventional 6-month threshold and the more recent definition proposed by the GCIG. According to the former, platinum resistance is identified as relapse occurring within 6 months following platinum-based chemotherapy [22], with patients experiencing relapse beyond this period considered platinum-sensitive. Additionally, we considered the treatment-free interval from the last platinum dose, measured in months from the time of the last chemotherapy to the onset of relapse.

The time to OS was measured in months from the time from diagnosis to the date of death or last follow-up.

### Measurement of Std KELIM

CA-125 concentrations were collected for each patient at diagnosis, before each cycle of chemotherapy, before IDS, and at the time of recurrence. The values corresponding to each cycle of chemotherapy were entered on the official KELIM site (https://www.biomarker-kinetics.org/CA-125-neo) to obtain the corresponding Std KELIM. Std KELIM is optimally calculated using at least three values for CA-125 during the first 100 days.

KELIM is derived from semi mechanical kinetic-pharmacodynamic modeling after logarithmic transformation of the CA-125 values. Mathematical modeling is performed with a nonlinear mixed effect model [21]. Std KELIM is calculated as follows: KELIM/cut-off defined by the Youden index in order to normalize the KELIM and to enable easy understanding of the threshold, according to You et al. [7]. A Std KELIM lower than 1 is considered unfavorable, while a Std KELIM is favorable. When Std KELIM was considered as discontinuous, the CHIVA tercile thresholds of 0.5 and 1 were also taken into consideration in the breakdown into 3 categories, i.e., unfavorable, intermediate and favorable. The thresholds for classifying KELIM into these same 3 categories (0.8 and 1.2), which were initially used on the biomarker-kinetics website, were also considered.

We carried out a sensitivity analysis on an optimal subgroup population: Std KELIM calculated with 3 CA125 values, with FIGO III/IV stage and with exclusion of histologies with low chemosensitivity (such as clear cells or mucinous).

### Platinum resistant recurrence score

You et al. defined the “Platinum-Resistant Recurrence Score” as a multivariate logistic regression model based on Std KELIM value and IDS completeness [11]. This model predicts the probability of subsequent platinum-resistant relapse.

We also evaluated the three prognostic groups described by Colomban in the post hoc ICON8, which combined achieving complete surgery and categorical Std KELIM: good if favorable KELIM (≥ 1) and complete surgery; intermediate if either unfavorable KELIM (< 1) or incomplete surgery; and poor if unfavorable KELIM and incomplete surgery. We evaluated the TFIp and OS for these group in our population.

### Statistical analysis

The statistical analyses were carried out with R software, version 4.0.2. The data are presented as mean ± standard derivation or number (n) with percentage. The Wilcoxon-Mann–Whitney test was used for the analysis of quantitative variables, and Fisher’s exact test was used for qualitative variables. We calculated the sensitivity, specificity, and positive and negative predictive values (PPV and NPV, respectively) of the Std KELIM.

The diagnostic accuracy of the test was measured by receiver operating characteristic (ROC) curve analysis and the area under the curve (AUC). The bootstrap method was used to calculate the 95% confidence intervals of the AUC.

Univariate and multivariate regression models were used to estimate the predictive value of Std KELIM.

The Platinum-Resistant Recurrence Score value was then used to construct a calibration curve of this score to evaluate its ability to predict platinum resistance in our population. Patients were grouped according to terciles of this score.

The prognostic value of Std KELIM for OS and TFIp was assessed with Kaplan‒Meier analysis and log-rank tests.

For all the statistical comparisons, a *p* value less than 0.05 was considered to indicate statistical significance.

## Results

### Patient characteristics

Out of 185 patients meeting the inclusion criteria, 152 patients (82%) had an abnormal baseline CA-125 level and a calculable Std KELIM and were included in the study. The clinical characteristics of patients whose KELIM could not be calculated were identical to those of the patients included in this analysis. Three patients were lost to follow-up at an early stage, resulting in a total of 149 patients available for analysis in our study.

The clinical and biological characteristics of the patients according to their platinum sensitivity status are presented in Table 1. Overall, 99 patients (66.4%) experienced platinum-sensitive recurrence, and 50 (33.6%) experienced platinum-resistant recurrence. The mean age was 63 years. Ninety-two percent of the patients had serous adenocarcinoma, and all had high-grade epithelial tumors according to final histology. The optimal IDS, i.e., CC0 status, was achieved in 60 of 149 patients (40%).

**Table 1:** Patient characteristics based on platinum-sensitivity status

***Characteristics***	***Overall Pop*** *(N* = *149)*	***Platinum Sensitive**** (N* = *99)*	***Platinum Resistant**** (N* = *50)*	***p value***
**Age (in years)**	63 (10)	63 (10)	63 (10)	0.90
**Histology**				0.92
Serous	137 (92.0%)	92 (93.0%)	45 (90%)	
Clear cell	3 (2.0%)	2 (2.0%)	1 (2.0%)	
Endometrioid	1 (0.7%)	1 (1.0%)	0 (0.0%)	
Mucinous	2 (1.3%)	0 (0.0%)	2 (4.0%)	
Undifferentiated	6 (4.0%)	4 (4.0%)	2 (4.0%)	
**FIGO stage**				0.60
IIB	2 (1.3%)	2 (2.0%)	0 (0.0%)	
IIIA	2 (1.3%)	2 (2.0%)	0 (0.0%)	
IIIB	2 (1.3%)	2 (2.0%)	0 (0.0%)	
IIIC	99 (66.0%)	67 (68.0%)	32 (64.0%)	
IVA	23 (15.0%)	14 (14.0%)	9 (18.0%)	
IVB	21 (14.0%)	12 (12.0%)	9 (18.0%)	
**Type of NAC**				0.40
VPlatinum and taxane	136 (91.3%)	93 (93.9%)	43 (86.0%)	
Platinum only	1 (0.7%)	1 (1.0%)	0 (0.0%)	
Other platinum-based NAC	12 (8.0%)	6 (6.1%)	7 (14.0%)	
**Number of Cycles of NAC**				0.40
< 3	1 (0.7%)	1 (1.0%)	0 (0.0%)	
3	4 (2.7%)	2 (2.0%)	2 (4.0%)	
4	35 (23.0%)	27 (27.3%)	8 (16.0%)	
5	9 (6.0%)	5 (5.1%)	4 (8.0%)	
6	83 (56.0%)	55 (55.6%)	28 (56.0%)	
> 6	17 (11.0%)	9 (9.1%)	8 (16.0%)	
**CA-125 at diagnosis**	1786 (2391)	1700 (2455)	1953 (2275)	0.11
**CA-125 before IDS**	85 (267)	41 (66)	175 (444)	** < 0.001**
**Std KELIM**	0.79 (0.38)	0.86 (0.37)	0.64 (0.35)	** < 0.001**
**Std KELIM**^**a**^				**0.004**
Unfavorable	109 (73%)	65 (66%)	44 (88%)	
Favorable	40 (27%)	34 (34%)	6 (12%)	
**RD after IDS**				**0.001**
No IDS	51 (34%)	33 (33%)	18 (36%)	
No CC0	36 (24%)	16 (16%)	20 (40%)	
CC0	60 (40%)	49 (49%)	11 (22%)	

Results are expressed in mean (standard deviation) or number of patients (percentage)

*CC0* absence of macroscopic postoperative residual disease, *NAC* Neoadjuvant chemotherapy, *IDS* Interval debulking surgery, *Pop* Population, *RD* Residual disease

^a^ Threshold used for these categories is 1

### Std KELIM

**Fig. 1:**
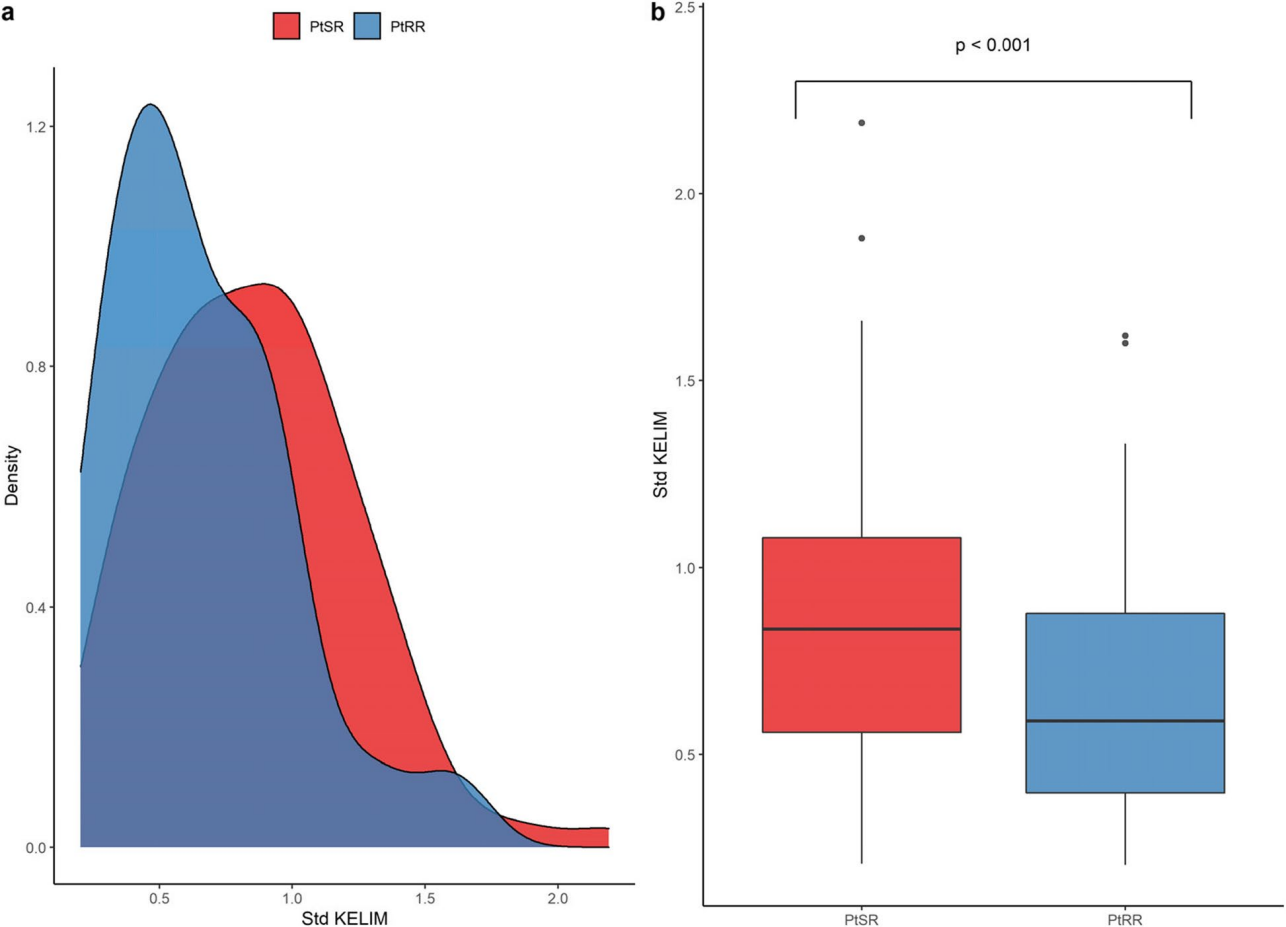
Distribution of Std KELIM according to platinum-sensitivity status (density curves (a) and boxplots (b))**.** PtSR: platinum-sensitive relapse / PtRR: platinum-resistant relapse. Mean Std KELIM was significantly lower in patients with PtRR versus PtSR patients at 0.64 vs 0.86, (*p* < 0.001)

The mean Std KELIM was significantly lower in patients who experienced platinum-resistant recurrence than in those who experienced platinum-sensitive relapse (0.64 vs 0.86, *p* < 0.001) (Fig. 1).

The mean pre-IDS CA-125 level was 41 IU/mL in the platinum-sensitive relapse group and 175 IU/mL in the platinum-resistant recurrence group. There was no significant difference in the baseline CA-125 concentration (1764 IU/mL vs 1725 IU/mL, *p* = 0.92) but significant difference on the CA-125 concentration before surgery (107 IU/mL vs 19 IU/mL, *p* < 0.004) between patients with a Std KELIM < 1 and patients with a Std KELIM score ≥ 1. The specificity and positive predictive value at this threshold were 94% and 95%, respectively. According to the univariate analysis, the CC0 score and CA-125 concentration just before IDS were the only two significantly different factors between the two groups (*p* < 0.001) (Table 1).

**Fig. 2:**
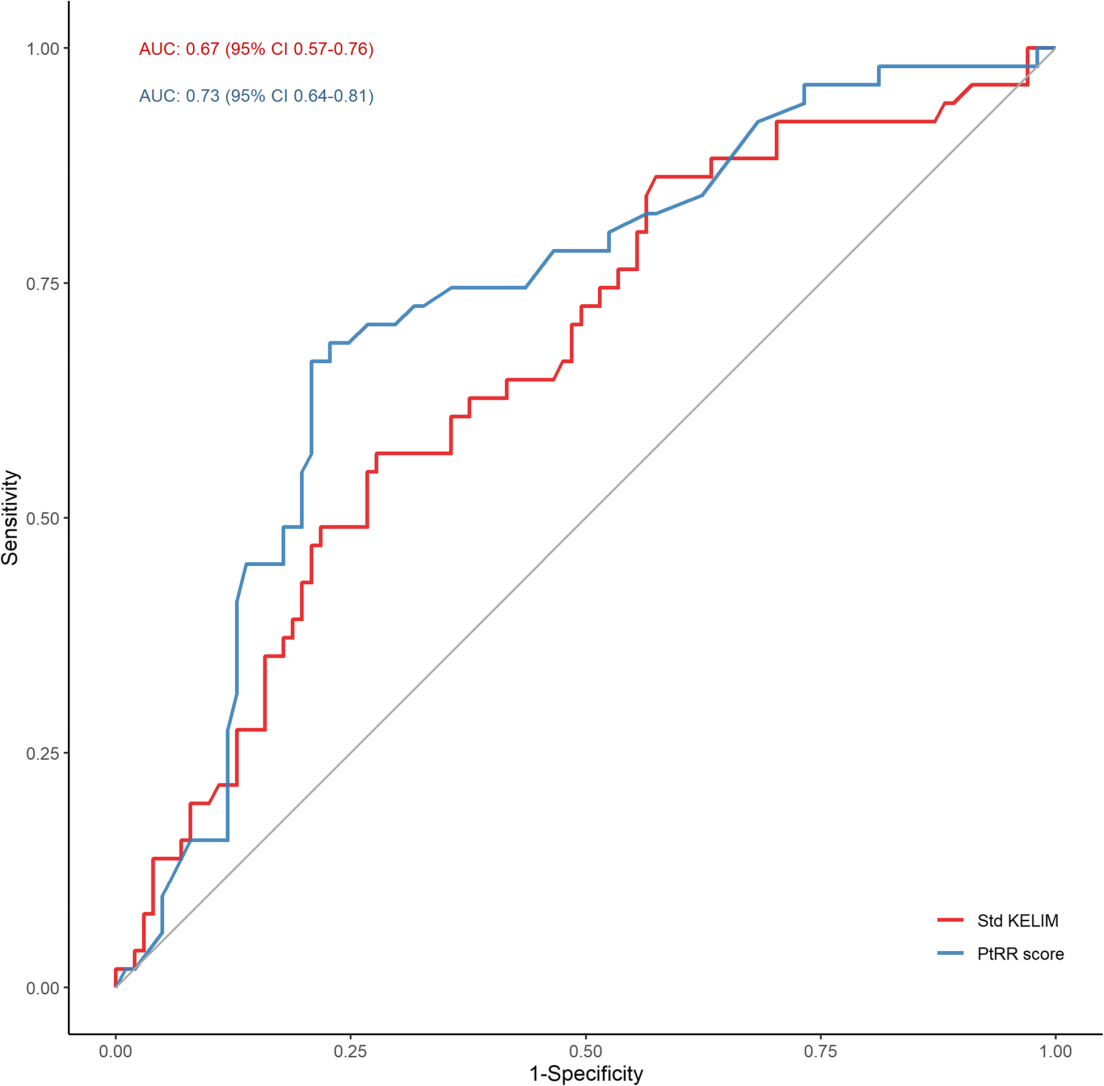
ROC curve for predictive value of Std KELIM and the platinum resistant recurrence score predicting platinum-resistant relapse. AUC: area under the receiver operating characteristic curve; CI: confidence interval; PtRR score: platinum resistant recurrence score

In our population, the discriminative ability of the Std KELIM regarding the PtRR was moderate, with an area under the ROC curve of 0.67 (95% CI [0.57, 0.76]). (Fig. 2).

We found a statistically significant association between favorable KELIM and the CRS3 chemotherapy response score (*p* = 0.038) but not with BRCA status (*p* = 0.78), but the analysis was limited by the availability of data (*n* = 79). An ANOVA was conducted to assess differences in Std KELIM according to chemotherapy regimen cycles. The results indicated no significant difference among the groups (*p* = 0.55).

We performed a sensitivity analysis on the subgroup of patients with an optimal KELIM calculation of stage FIGO III/IV, excluding those with low-chemosensitivity histologies (such as clear cells or mucinous). A total of 109 patients met these criteria. The discriminative ability was almost identical, with an AUC of 0.70 (95% CI [0.60, 0.80]).

The robustness of Std KELIM to the different thresholds used during Std KELIM development was also assessed. The thresholds used in CHIVA (0.5;1) and in the first version of the online tool (0.8;1.2) determined 3 groups (unfavorable, intermediate, favorable). Regardless of the thresholds chosen, Std KELIM remained statistically significant, demonstrating the robustness of Std KELIM (Table 2).

**Table 2:** Exploration of the historic different thresholds defined in the study by You et al. and in our study

**Std KELIM threshold**	***Overall Pop*** *(N* = *149)*	***Platinum Sensitive*** *(N* = *99)*	***Platinum Resistant*** *(N* = *50)*	***p value***
**CHIVA Tercile**				
** Unfavorable (< 0.5)**	38 (25.5%)	17 (17.2%)	21 (42.0%)	**0.001**
** Intermediate (0.50–1.0)**	71 (47.7%)	48 (48.5%)	23 (46.0%)	
** Favorable (> 1.0)**	40 (26.8%)	34 (34.3%)	6 (12.0%)	
**First Tercile**				
** Unfavorable (< 0.8)**	84 (56.4%)	48 (48.5%)	36 (72.0%)	**0.022**
** Intermediate (0.8–1.20)**	44 (29.5%)	35 (35.4%)	9 (18.0%)	
** Favorable (> 1.20)**	21 (14.1%)	16 (16.1%)	5 (10.0%)	

According to the univariate analysis, Std KELIM was a significant predictor of platinum resistance recurrence, regardless of whether it was considered a continuous covariate, where the odds ratio (OR) was 0.19 (95% CI [0.06, 0.53] *p* = 0.002), or a discrete covariate (*p* < 0.004). The lack of completeness of IDS was also a significant covariate in the univariate analyses, with an OR for CC1 + versus CC0 of 4.64 (95% CI [2.27, 9.75] *p* < 0.001). However, residual disease after IDS was the only independent predictor in the multivariate regression model, with an OR of 3.6 (95% CI [1.6, 7.9] *p* = 0.001).

**Fig. 3:**
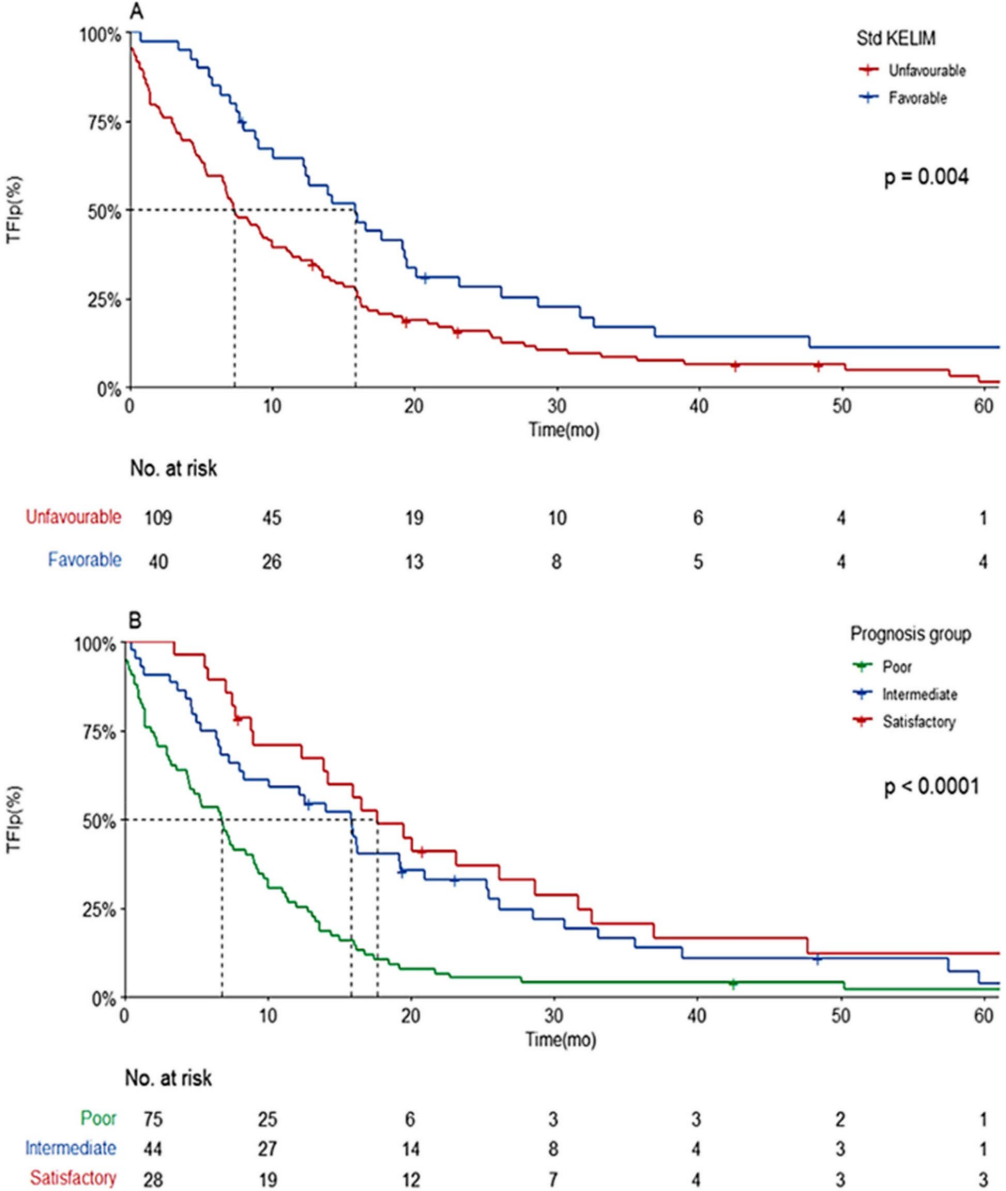
Prognosis value of Standardized KELIM score regarding TFIp and OS according to Standardized KELIM Score and Prognosis group. **A** Kaplan–Meier curve of TFIp according to Std KELIM Score (**B**) Kaplan–Meier curve of TFIp according to the three-prognosis group based on Std KELIM score and completeness of IDS

Our population had a median overall survival of 43.1 (95% CI 39.9–55) months and a TFIp of 9.18 (95% CI 7.38–12.8) months. A favorable Std KELIM score (≥ 1) was associated with a better median TFIp (15.9 vs 7.3 months, *p* = 0.004) than was an unfavorable Std KELIM (Fig. 3).

By combining the 2 components of the platinum resistance score (Std KELIM used in its dichotomous form and surgical completeness status), 3 prognostic groups were defined: good prognosis (favorable kelim and complete surgery), poor prognosis (unfavorable kelim and incomplete surgery) and intermediate prognosis (one of the unfavorable components), according to post hoc analysis of ICON8 [24]. These 3 groups exhibited statistically significant differences in terms of OS and TFIp. The group with poor prognosis had a median TFIp of 6.8 months compared to 15.8 and 17.7 months for the intermediate and good prognosis groups, respectively (Fig. 3).

### Platinum resistant recurrence score

The ROC curve for the Platinum Resistant Recurrence Score showed good accuracy, with an AUC of 0.73 (95% CI [0.64, 0.81]) (Fig. 2).

**Fig. 4:**
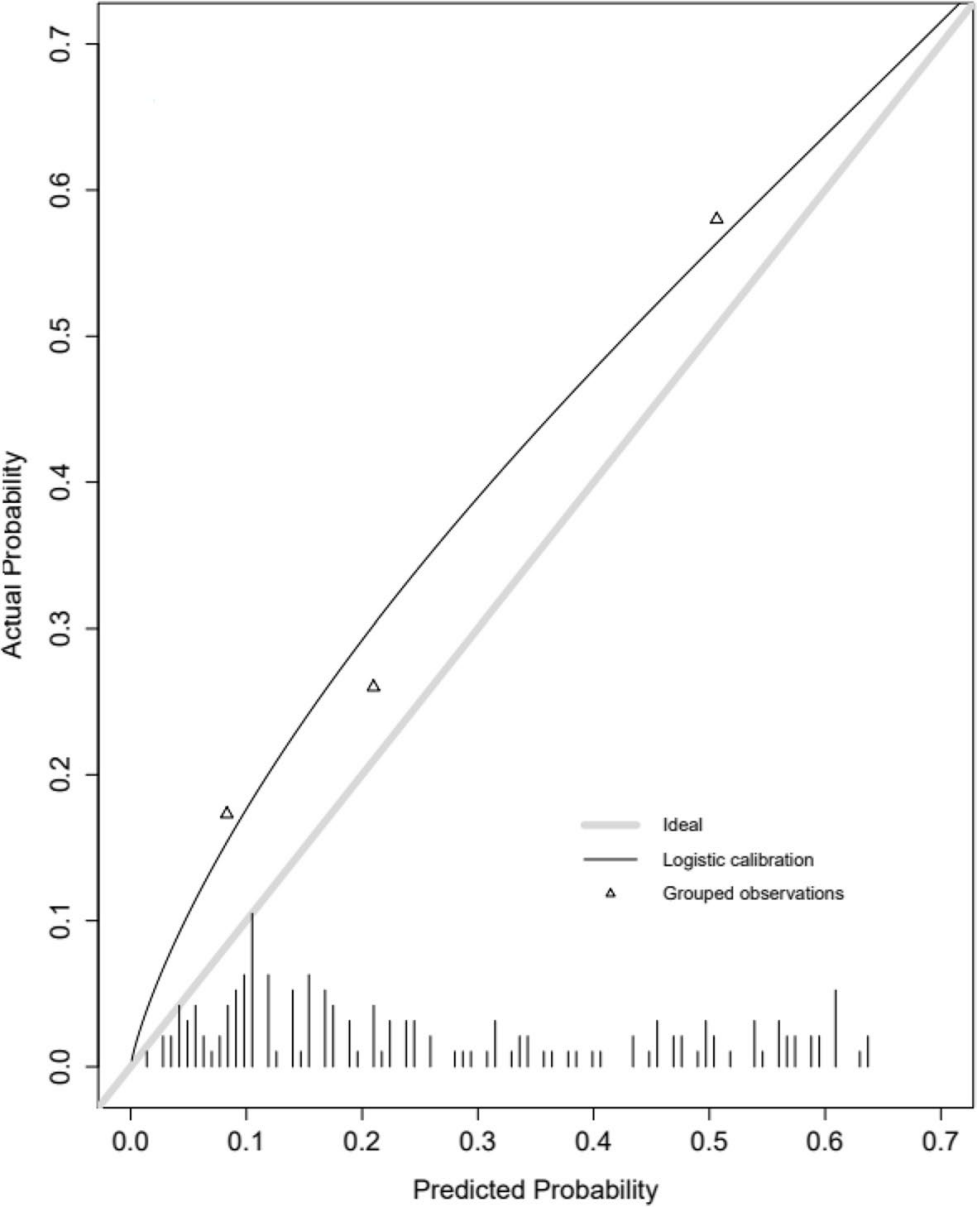
Calibration curve of the Platinum resistant Recurrence Score. The grey line “ideal” corresponds to perfect calibration. The black line is the logistic calibration of the platinum resistant recurrence score, according to the completeness of interval debulking surgery status. The grouped observations, three triangles, are the terciles of our population

A calibration analysis of the Platinum Resistant Recurrence Score was also performed, considering, for each patient, the probability of platinum-resistant relapse corresponding to her IDS status, i.e., CC0 or no CC0. The calibration curve obtained showed a slight but significant underestimation (*p* = 0.02) of the risk of platinum-resistant relapse (Fig. 4).

This underestimation was present for the three terciles of our population represented. The mean absolute error in the predicted probabilities was 7.1%, and the 0.9 quantile of the absolute errors was 14.3%.

## Discussion

To date, few studies have evaluated the role of CA-125 in predicting platinum sensitivity. For patients receiving NAC, different cut-offs have been published, ranging from 35 IU/mL before IDS [12, 19] or after the 3rd cycle of NAC [14] to 200 IU/mL before IDS [16], or an 80% decrease in CA-125 concentration [17, 18]. Modern approaches based on mathematical modeling, including Std KELIM, are promising because they allow for earlier prediction of the chemosensitivity status. At least three available CA-125 values during the first 100 days of neoadjuvant chemotherapy are required to ensure accurate assessment of Std KELIM by the model. In our study, high specificity and positive predictive value were particularly promising, indicating that Std KELIM may be a useful tool for decision-making by clinicians as early as the third cycle of NAC.

Defining the optimal threshold for Std KELIM is a major prerequisite for future clinical decision-making and was heterogeneous according to earlier reports [25]. Other thresholds may be considered to favor the sensitivity or the specificity of the prediction. In the present case, optimal sensitivity and negative predictive value may be preferable to identify the maximum number of patients at risk of platinum-resistant relapse to propose appropriate alternative treatment.

Our study also evaluated the Platinum-Resistant Recurrence Score, which, by its fair calibration, could also be an effective early tool for decision-making on treatment adaptation, as the mean error (7.1%) is compatible with its use in routine practice. The score could be particularly useful for triage in decision making. With this objective of very early prediction, Bouvarel et al. published and validated the use of the neoadjuvant Std KELIM as a major independent predictor of the probability of complete surgery and survival [26]. Their findings, along with our results for the three prognostic subgroups, reaffirm that both the primary chemosensitivity of the tumor and the completeness of surgery are significant and complementary prognostic factors. This complementarity is echoed in the current definition of platinum sensitivity, which is recognized to correlate with sensitivity to PARP inhibitors as well. Some authors propose that when prescribing maintenance treatments, first-line therapeutic adjustments could be guided by these subgroups [24, 27]. However, balance should be struck in this approach, as a meta-analysis conducted by Corbaux et al. [28] highlighted only moderate predictive capacity but rather good prognostic capacity of Std Kelim. Notably, this analysis did not identify KELIM as a potential surrogate marker for progression-free survival (PFS) or overall survival (OS).

More data and more complex models are needed to better understand the interactions between clinical and biological parameters associated with platinum sensitivity prediction. For example, pathological response after NAC may be a useful discriminating factor for predicting platinum sensitivity. The chemotherapy response score (CRS) [28] stratifies patients into complete/near-complete (CRS3), partial (CRS2), and no/minimal (CRS1) response groups after NAC. In women with high-grade serous tubo-ovarian carcinoma treated with NAC, CRS3 was significantly associated with improved PFS and OS. Consideration of CRS or the use of other pathophysiological assessment models in a predictive model of platinum sensitivity after NAC appears to be a promising approach [29]. Marchetti's recent ASCO publication revealed that favorable KELIM was significantly associated with CRS3 [30]. This finding should indeed be confirmed in a new study to assess its predictive effect in association with CC0, homologous recombination deficiencies (HRD) and KELIM [31]. Other potential predictive markers, such as RECIST 1.1 radiological response or the genomic status of the patient, also deserve to be investigated in future studies. Bogani et al. showed that the RECIST 1.1 response criterion may be helpful for predicting surgical resectability and disease-free survival in patients with advanced EOC who are receiving neoadjuvant chemotherapy plus IDS (HR 0.42 95% CI [0.09, 0.78] *p* = 0.001) [31].

Genomic status and other biomarkers, such as BRCA mutational status or HRD status, and microRNAs involved in the homologous recombination pathway may also exhibit predictive value [32]. Tumiati et al. showed that low HR scores predicted primary platinum sensitivity with high statistical significance (*p* = 0.01) [33]. Pennington et al. also showed that the presence of germline and somatic HR mutations was highly predictive of primary platinum sensitivity (*p* = 0.0002) [34]. These studies indicate the need to take genomic status into account in future studies

Our study has several limitations that we may acknowledge. Our overall population was more heterogeneous, particularly with regard to FIGO stage and the number of cycles of NAC, than those in earlier studies. This heterogeneity may have influenced the results in our study compared to the CHIVA population used to determine the optimal threshold of Std KELIM. We have chosen not to exclude unconventional clinical interventions to faithfully emulate real-life settings. This methodology facilitates the evaluation of KELIM's robustness as an early independent prognostic marker, irrespective of potential fluctuations in treatment strategies, such as the use of monotherapies due to treatment-induced toxicities or patient frailty.

Moreover, our study was retrospective in nature. Only 29% of the population received 3 or 4 courses of chemotherapy, and only a minority of patients (5.9%) received antiangiogenic therapy in the neoadjuvant setting, in contrast to the results of the You et al. study [11]. The CHORUS trial was published after the inclusion period of our study. At that time, Kang's meta-analysis showed no impact of the number of NAC cycles with an increased rate of optimal cytoreduction, which explains the classic approach of 6 courses of chemotherapy [35]. Until 2010, the main chemotherapy protocol chosen was 6 cycles of neoadjuvant chemotherapy, with a gradual increase in 2011 to 4 cycles of chemotherapy with surgery in between. This regimen became the majority from 2013 onwards.

The heterogeneity of our study population stems from the evaluation of patients treated outside of a clinical trial, in contrast with most validation studies of Std KELIM, which were mainly carried out on clinical trial databases. Despite this difference, our approach and findings offer valuable insights and confirmed the prognostic aspect of the Std KELIM and its good predictive quality. First, it supports Std KELIM's utility in real-world settings, particularly when CA125 sampling times are more varied than in clinical trials. Second, they indicated that Std KELIM remains a marker of chemosensitivity even when neoadjuvant chemotherapy extends beyond three cycles before debulking. These findings suggest that prolonged chemotherapy in the neoadjuvant setting does not solely lead to platinum salt resistance. Third, our sensitivity analysis of the subpopulations (advanced stage, comparison to CRS, etc.) suggested that Std KELIM's information is partially complementary.

Interestingly, rapid advances in treatment regimens and the advent of new therapeutic approaches may involve the use of Std KELIM and PtRR scores to enhance precision and personalized medicine implementation in comparison to predictions with standard or innovative treatments.

## Conclusion

The Std KELIM and PtRR score may be a future aid to clinicians to identify those patients who will have the highest probability of platinum-resistant relapse after IDS and a poorer prognosis. The implementation of new measures in the adjuvant setting can then be considered. However, further studies considering new markers of chemosensitivity and the use of innovative drugs are needed.

### Supplementary Information

Supplementary Material 1.

## Abbreviations

AUC: Area under the curve
CC score: Completeness of cytoreduction score; CC0: No residual disease
EOC: Epithelial ovarian cancer
FIGO: International Federation of Gynecology and Obstetrics
IDS: Interval debulking surgery
NAC: Neoadjuvant chemotherapy
NPV: Negative predictive value
OR: Odds ratio
PFI: Platinum-free interval
PtRR: Platinum-resistant relapse
PtSR: Platinum-sensitive relapse
PPV: Positive predictive value
ROC: Receiver operating characteristic
YI: Youden index
YP: Youden point
