# Supplementary material for: External validation of Standardized KELIM and platinum-resistant recurrence scores in patients with advanced epithelial ovarian cancer
Source: J Ovarian Res. 2024 Jul 22;17:152. doi: 10.1186/s13048-024-01476-3 (PMC11265035; doi:10.1186/s13048-024-01476-3)
Supplement: Supplementary file 1 — Supplementary Material 1. [file 13048_2024_1476_MOESM1_ESM.docx]

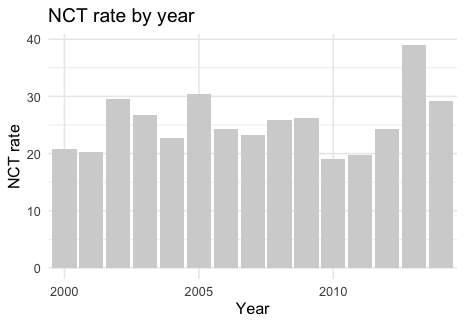


*Supplementary Material 1: Evolution of chemotherapy rates for advanced-stage ovarian cancers over the study period*
